# Passive Microwave Radiometry and microRNA Detection for Breast Cancer Diagnostics

**DOI:** 10.3390/diagnostics13010118

**Published:** 2022-12-30

**Authors:** Leonid Fisher, Olga Fisher, Dmitry Chebanov, Sergey Vesnin, Alexey Goltsov, Arran Turnbull, Mike Dixon, Indira Kudaibergenova, Batyr Osmonov, Sergey Karbainov, Larion Popov, Alexander Losev, Igor Goryanin

**Affiliations:** 1 Russian Academy of Medico-Social Rehabilitation, Moscow 105037, Russia; 2 BioAlg Corp., Walnut, CA 91789, USA; 3 Medical Microwave Radiometry (MMWR) Ltd., Edinburgh EH10 5LZ, UK; 4 Institute for Artificial Intelligence, Russian Technological University (MIREA), Moscow 119454, Russia; 5 Edinburgh Cancer Research, University of Edinburgh, Edinburgh EH8 9YL, UK; 6 Kyrgyz State Medical Academy (KSMA), Bishkek 720020, Kyrgyzstan; 7 Aragon Group Inc., Aeulestrasse 30, 9490 Vaduz, Liechtenstein; 8 Faculty of Mathematics and Information Technology, Volgograd State University, Volgograd 400062, Russia; 9 Biological Systems Unit, Okinawa Institute Science and Technology Graduate University, Okinawa 904-0495, Japan; 10 School of Informatics, University of Edinburgh, Edinburgh EH8 9AR, UK

**Keywords:** breast cancer, early diagnostics, passive microwave radiometry (MWR), microRNA (miRNA)

## Abstract

Breast cancer prevention is an important health issue for women worldwide. In this study, we compared the conventional breast cancer screening exams of mammography and ultrasound with the novel approaches of passive microwave radiometry (MWR) and microRNA (miRNA) analysis. While mammography screening dynamics could be completed in 3–6 months, MWR provided a prediction in a matter of weeks or even days. Moreover, MWR has the potential of being complemented with miRNA diagnostics to further improve its predictive quality. These novel techniques can be used alone or in conjunction with more established techniques to improve early breast cancer diagnosis.

## 1. Introduction

The main goal of breast cancer screening is to detect the cancer at the earliest possible stage. The modern diagnostic methods of mammography (MMG), ultrasound, computer tomography (CT), and magnetic resonance imaging (MRI) can detect tumors as small as 3 mm in diameter.

The classification of breast cancer pathology is based on the Breast Imaging Reporting and Database System (BI-RADS) score, which is used by clinicians and radiologists to describe mammogram results [1]. It has been shown that the majority of breast cancer-related deaths are associated with aggressive, fast-growing cancers [2], and these fall into the category transit BI-RADS-3; this category is therefore of particular importance.

Clinical recommendations state that a follow-up mammogram should be done within 3–6 months of initial diagnosis. Most of the time, BI-RADS-3 cancers change into BI-RADS-2, which is considered a benign process. Unfortunately, after passive observation, 2–4% of these cases then progress to BI-RADS-4a, indicating a risk of cancer of up to 10%. As a result, many BI-RAD-4a breast cancers are only detected after 3–6 months, thereby preventing these patients with aggressive breast cancer from receiving timely treatment. The consequence of this is an increase in mortality. Novel diagnostic techniques that would permit the identification of potentially harmful BI-RAD-3 tumors would allow us to foresee the development of aggressive BI-RADS-4 cancer and, ultimately, to reduce breast cancer-related deaths.

The purpose of this study was to assess the potential of two new screening methods, passive microwave radiometry (MWR) and miRNA detection, to identify BI-RADS-3 cases. MicroRNAs (miRNAs) are small, non-coding RNA molecules. With their tissue-specific expression, correlation with clinicopathological prognostic indices, and known dysregulation in breast cancer, miRNAs have quickly become an important avenue in the search for novel breast cancer biomarkers [3,4,5].

## 2. Materials and Methods

We first examined 230 patients aged 35–55 years (mean age ± standard deviation: 43 ± 2.4 years) by MMG (AMULET; Fujifilm, Tokyo, Japan) to ascertain BI-RADS scores. The scans were taken in standard craniolateral and mediolateral projections. The patients were then examined by ultrasound (DC-60Exp; MindRay, China), Microwave Radiometry which measures internal radiation from human bodypassively, WR (MWR2020 [formerly RTM-01-RES]; Medical Microwave Radiometry Ltd., Edinburgh, UK) [6,7], and an miRNA oncopanel (Oncounite; Skolkovo, Russia) [8]. The follow-up period was 12 months.

**Figure 1:**
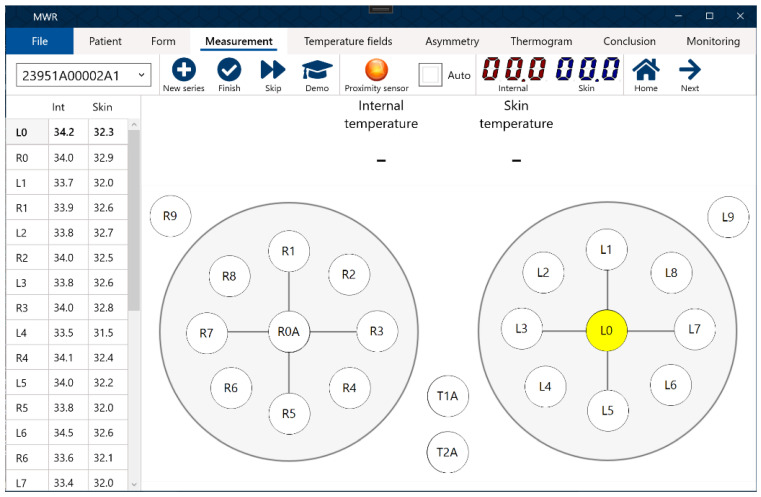
Interface of the MWR2020 software. Measurement points are shown.

**Figure 2:**
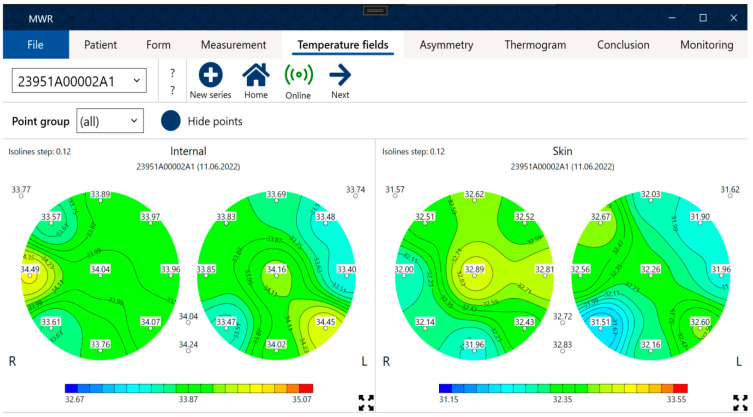
Interface of the MWR2020 software. Temperature measurements of a healthy patient.

Using the MWR2020 device, internal and skin temperature measurements were conducted at 22 points (left and right breast). Control points are shown in Figure 1. The results were visualized (Figure 2) and stored in CSV data format for further analysis.

For further assessment, the temperatures were combined into groups:

- Skin temperature of the right breast:

(1) $$T^{r,ir}=\{t_{0,r}^{ir},\ldots ,t_{9,r}^{ir}\}$$

- Skin temperature of the left breast:

(2) $$T^{l,ir}=\{t_{0,l}^{ir},\ldots ,t_{9,l}^{ir}\}\;$$

- Skin temperature of the body (reference points T1 and T2 in Figure 1):

(3) $$T^{a,ir}=\left\{t_{1,a}^{ir},\ldots ,t_{2,a}^{ir}\right\}$$

- Internal temperature of the right breast:

(4) $$T^{r,mw}=\{t_{0,r}^{mw},\ldots ,t_{9,r}^{mw}\}\;$$

- Internal temperature of the left breast:

(5) $$T^{l,mw}=\{t_{0,l}^{mw},\ldots ,t_{9,l}^{mw}\}$$

- Internal temperature of the body at control points:

(6) $$T^{a,mw}=\left\{t_{1,a}^{mw},\ldots ,t_{2,a}^{mw}\right\}$$

For temperature *t*, superscripts *ir* and *mw* indicate skin and internal temperature spectrums, respectively. The first subscript value is the number of the survey point to which the temperature value belongs (the number corresponds to the examination scheme in Figure 1), and the second value indicates to which of the paired organs the temperature belongs (*r*, right; *l*, left).

We used two empirical coefficients [9] to stratify and divide the patients into three groups depending on the assessed risk of developing breast cancer. The average internal and skin temperatures of the breast tissue were calculated. The difference between the maximum (7), (8) and average skin and internal temperatures for each gland (9), (10) were also calculated. The obtained differences were summarized for the right and left breasts:

(7) $$T^{max,\;mw}=\max\left(\left\{T^{r,\;mw},T^{l,\;mw}\right\}\right)$$

(8) $$T^{max,\;ir}=\max\left(\left\{T^{r,\;ir},T^{l,\;ir}\right\}\right)$$

(9) $$T^{mean,\;mw}=\bar{\left\{T^{r,\;mw},T^{l,\;ir}\right\}}$$

(10) $$T^{mean,ir}=\bar{\left\{T^{r,\;ir},T^{l,\;ir}\right\}}$$

The largest value of $Q_{max}$ was chosen by

$$Q_{max}=\max\left(\left(T^{max,\;mw}-T^{mean\_mw}\right),\;\left(T^{max,\;ir}-T^{mean,\;ir}\right)\right)$$

Next, the maximum temperature difference between separate symmetrical points of the left and right mammary glands ($k_{int},\;\mathrm{internal}$; $k_{skin},\;\mathrm{skin}$) were determined by

(11) $$k_{int}=\max\left(T^{l,\;mw}-T^{r,mw}\right)$$

(12) $$k_{skin}=\max\left(T^{l,\;ir}-T^{r,ir}\right)$$

From these values, the parameter R was calculated. If the maximum temperature difference for both sensors corresponded to the same point on the measurement scheme, R was calculated as the sum of $k_{int}\;$ and $k_{skin}$*:*

(13) $$R=k_{int}+k_{skin}$$

Otherwise, the maximum value is selected among the parameters *k_int_* and *k_skin_*, and the difference of symmetrical temperatures at the point (parameter n) at which the maximum value is taken between these parameters is added to it. In this case, the difference is calculated by the second sensor (parameter x), i.e., if the maximum value was by the sensor that measures skin temperatures, the difference in deep temperatures is added to it:

(14) $$R=\max\left(k_{int},k_{skin}\right)+(t_{n,l}^{x}-\;t_{n,r}^{x})$$

For cases where $Q_{max}\;$> 2.0 and R > 2.5, a malignant tumor was suspected. Conversely, if $Q_{max}\;$< 2.0 and R < 2.5, a benign tumor was diagnosed. If either $Q_{max}$ or R exceeded these thresholds, both malignant and benign tumors were possible. 

Simultaneously with the described method, to determine the risk group according to microwave radiometry, artificial intelligence methods were used, in particular, the weight agnostic neural network, configured by the bi-population covariance matrix adaptation evolution strategy method [10]. The main feature of this neural network is that at the stage of searching for the optimal network architecture for the task, the average weights of the model are used. Thus, a high convergence of the neural network optimal architecture selection is achieved. The neural network was trained on a sample of 4377 low-risk breast cancer patients and 535 high-risk patients. The feature space built on the basis of the conceptual model described in [11] was used as the input layer of the neural network. At the output, the neural network gave out whether the patient belongs to a high-risk group of breast cancer. As a result of computational experiments, the F1-score of the neural network reached 0.933 when cross-validated on the described database of low- ang high- risk breast cancer patients.

**Figure 3:**
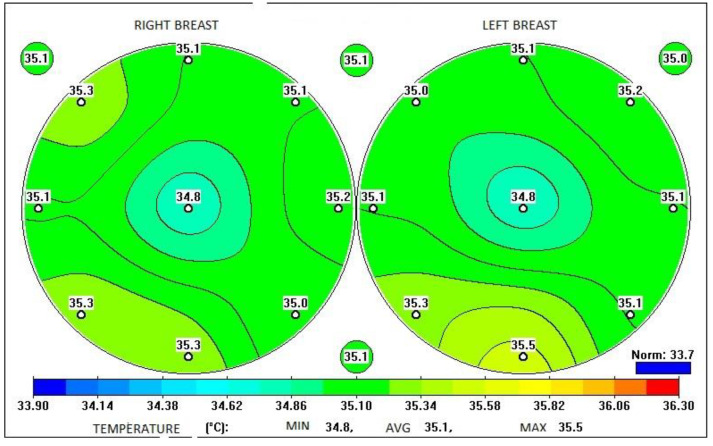
Interface of MWR2020 software. Temperature measurements of a patient with a high probability of breast cancer.

This conceptual model describes the various characteristic features of thermograms of patients with and without breast cancer. The thermograms in Figure 3 of a patient with a high probability of developing cancer display the following features:

- High spread of skin temperatures (more than 2 °C);
- An area of significantly elevated temperature in the left breast;
- The same area shows a reduced difference between skin and internal temperatures;
- High difference between the skin temperatures of symmetrical measurement points of the breasts.
- In the left mammary gland, the area of elevated skin temperature (the prevalence of the proliferative process) is 23%, while in the right mammary gland, the temperature increase is practically missing.

In contrast, the thermograms shown in Figure 2 of a healthy patient do not display any of these features; no areas with elevated temperatures are observed, and temperature fluctuations are within the normal range. Note that not all characteristics of patients at risk of developing breast cancer are described here. For example, patients also commonly have specific areas with elevated temperatures, such as in the nipple area.

Based on these irregularities, a feature space was built. To do this, the measured temperatures were divided into groups (described earlier by (1), (2), (4), and (5)) and their ratios were determined by the following:

- Internal temperature gradient of the right mammary gland:

(15) $$T^{r,g}=T^{r,mw}-T^{r,ir}$$

- Internal temperature gradient of the left mammary gland:

(16) $$T^{l,g}=T^{l,mw}-T^{l,ir}$$

- Thermal asymmetry according to skin temperatures:

(17) $$T^{ta,\;ir}=T^{l,\;ir}-T^{r,ir}$$

- Thermal asymmetry according to internal temperatures:

(18) $$T^{ta,mw}=T^{l,mw}-T^{r,mw}$$

Here, the internal gradient is the difference between the deep internal and skin temperatures measured at the same point of examination. Thermal asymmetry is the difference between temperatures at symmetrical measurement points of the right and left mammary glands.

Various operations were applied to these groups and, as a result, signs characteristic of certain temperature anomalies were obtained. For example, the oscillation of the skin temperature of a mammary gland characterizes the spread of temperatures in the gland, and the maximum of these temperatures characterizes the presence of hot areas. The feature space constructed in this way increased the accuracy of the neural network by 1% [12].

A novel miRNA panel was used [8] to assess the risk and dynamics of breast cancer development. The panel includes measurements of eight types of miRNA:

1. Hsa-miR-199a-3p—causes metastasis by stimulating angiogenesis and tumor progression through overexpression of ApoE.
2. Hsa-miR-222-3p—suppressor of apoptosis in cells, resulting in increased proliferation as well as differentiation. Is increased in at-risk individuals.
3. Hsa-let-7a-5p—increases levels of integrin B-3, which is associated with tumor necrosis factor and the control of proliferation. Indicates a reduced risk of disease.
4. Micro-RNA-196a-2—a protective factor that reduces tumor growth and proliferation, as well as controlling cellular migration and invasion. Reduces the risk of tumor formation.
5. Hsa-miR-106a-5p—induces apoptosis and reduces proliferation. Is reduced in tumors.
6. Hsa-miR-21-5p—activates the PI3K/Akt pathway to increase cell proliferation and survival. Is increased in at-risk individuals.
7. Hsa-miR-21-137—regulates proliferation and apoptosis. Is reduced in at-risk individuals.
8. Hsa-miR-155—causes a decrease in taurine levels, increasing the effect of oxidative stress. Is increased in individuals with taurine deficiency.

Isolation of miRNA was carried out according to the following method: Proteinase K was added to the patient samples to a concentration of 5 μM and incubated at 56 °C for 1 h. The samples were applied to Exigon columns (Exigon, Huldenberg, Belgium) and centrifuged at 2000× *g* in an Eppendorf 5104 centrifuge (Eppendorf, Hamburg, Germany). The columns were then washed with washing buffer and centrifuged again at 2000× *g*. This washing procedure was repeated three times. Reverse transcription was performed by adding 8 μL of reverse transcriptase to 8 μL of sample and incubating the mixture for 1 h at 60 °C. Real-time polymerase chain reaction (qPCR) was performed using an Applied Biosystems 7500 Real-Time PCR System (Thermo Fisher Scientific, Waltham, MA, USA) with primers for the following miRNAs: hsa-miR-199a, hsa-miR-214-3p, miR-25, miR-26a, hsa-let-7a-5p, miR-99a, miR-184, miR-24-3p, miRNA-195, hsa-miR-21-5p, and hsa-miR-195. The results were analyzed using GenEx qPCR software (MultiD Analyses AB, Gothenburg, Sweden).

## 3. Results 

**Figure 4:**
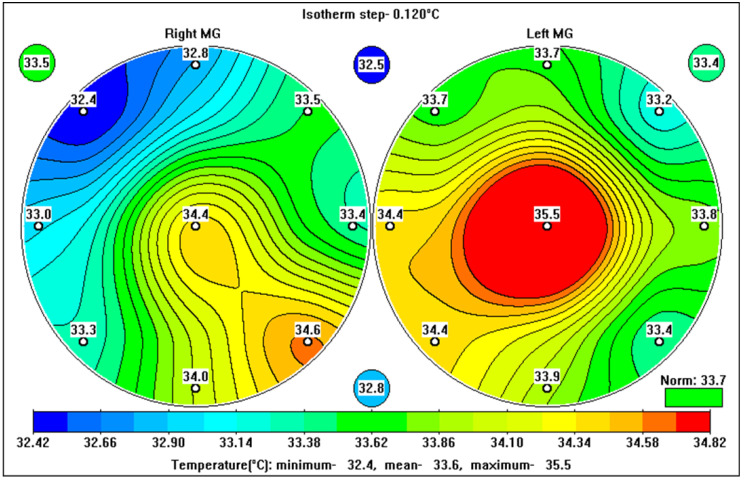
Internal temperature fields in the left (**right**) and right (**left**) mammary glands of an individual at risk of developing breast cancer.

MMG exams were performed two times over the course of a year. Three patients showed an increase in tumor density (>5) and were transferred to the BI-RADS-4a category. We found a positive correlation between an increase in BI-RADS and increases in the *Q_max_* and R coefficients (Figure 4). Moreover, an increase in BI-RADS correlated significantly with an increase in oncological miRNA. For those patients where there was no increase in radiological density, there were no negative dynamics associated with MWR or the miRNA oncopanel.

From morphological studies of punctures in patients with an increase in BI-RADS, it was found that high MWR coefficients (*Q_max_* > 2.0 and R > 2.5) and miRNA changes (>5) indicated a risk of developing obligate precancer, where the tumor never progresses into any type of lesion other than cancer. These results were confirmed by in situ histological parameters in two cases. 

### Clinical Example

Patient P., born in 1957, had complained of scanty discharge from the nipple of the right breast. MMG showed a picture of pronounced adenosis, with a high radiographic density of 5. Ultrasound imaging of the mammary glands, which showed no nodular formations, revealed many microcysts with a diameter of 4–5 mm. MWR examination of the mammary glands showed that *Q_max_* was elevated (1.7; the threshold is 2) and that R was slightly elevated (1.9; the threshold 2.5). The patient was also classified by the neural network, which identified the patient as being in the high-risk group of developing breast cancer. To further clarify the diagnosis, the patient’s levels of miRNAs in the oncopanel were determined (Table 1). The results of the panel indicated that the patient’s risk of developing breast cancer was slightly increased to a risk factor of 3 (1 + 1 + 1), which is typical for patients with a low risk of developing breast cancer (Table 2).

After 6 months, the patient went for a follow-up examination with complaints about the appearance of a nodular formation in the right mammary gland and enlarged axillary lymph nodes on the right. MMG determined a nodular polycyclic formation of 20 mm in diameter in the right mammary gland and the presence of grouped microcalcifications. Ultrasound of the right mammary gland showed an irregularly shaped quadrant hypoechoic formation of 20 mm and enlarged nodes of up to 25 mm in the right axillary region. MWR assessment showed *Q_max_* and R values of 2.45 and 3.2, respectively, which indicated a probability of developing breast cancer of over 85%. The results of the dynamic study of miRNAs are shown in Table 3.

The risk factor was 7 (3 + 3 + 1), which is typical of patients with a high risk of developing breast cancer. Histological examination confirmed the presence of invasive carcinoma.

## 4. Discussion

We propose a new scientific approach to breast cancer screening that utilizes the biophysical and molecular biology principles of MWR and miRNA analysis. This novel approach could be used to supplement existing established methods, such as MMG and ultrasound. 

In several clinical examples [13] using two coefficients [9], data obtained using MWR showed to be ahead of MMG results by 1.5–2 years. More recently, artificial intelligence-based approaches have been applied to MWR data for early stage breast cancer prediction to approve diagnostic accuracy. For example, in a study on MWR data from >4000 patients who had been classified by clinicians as either being at low or high risk of developing breast cancer, deep neural networks achieved with an accuracy of breast cancer prediction >0.93 [10,14,15].

Despite the advantages afforded by MWR, the technique has certain limitations in some pathologies of the mammary gland. First, with slow growing malignant neoplasms, heat dissipation can be masked by the heat generated by the surrounding tissues. Second, a large malignant neoplasm of the mammary gland (40–50 mm) can be limited to a fibrous capsule, which, like a Dewar’s vessel, does not allow heat to go beyond the border of the tumor. These problems can be largely solved with more frequent measurements, such as over the course of a few weeks or even days. Other methods of thermometry could also be explored for cancer detection [16,17].

Another novel way in which the pathology of breast cancer tissue could be assessed is to analyze the ratio of disease markers [18]. Since genetic mutations in precancerous and neoplastic pathologies are of the same nature, the factors that are indicative of the qualitative transition from precancerous pathology to the neoplastic process could be identified by determining the mutations in precancerous neoplasms. While this method is quite promising in relation to the administration of targeted therapy, it is costly and impractical.

Another solution is the analysis of intercellular interactions, i.e., the result of mutant genes. It has been shown that exosomes and, more specifically, their qualitative composition play a leading role in this process. Cellular communication through exosomes is able to influence the fate of cells when under stress, such as exposure to ionizing radiation. In vitro and in vivo studies have shown that exosomes may play a role in off-target radiation effects by carrying molecular signaling mediators of radiation damage, as well as performing protective functions that lead to resistance to radiation therapy. Moreover, miRNA expression is affected by tissue exposure to radiation, and exosomes from the plasma of irradiated mice prevent radiation-induced apoptosis [19]. Thus, the main factor that transmits information is the quantitative and qualitative miRNA composition of exosomes.

miRNAs are the “global switches of the genome”, regulating multiple metabolic pathways and the formation of protein products. Some miRNAs, including 21, 155, 196a-2, 27a, 9, 199a-3p, 222-3p, let-7a-5p, 137, and 106a-5p are known to have oncogenic effects [20]. miRNA-21 is one of the most well-known miRNAs and has been studied extensively in different types of tumors. Its expression, which sharply increases in breast cancer, causes apoptosis genes to be blocked and is therefore associated with tumor growth, metastases, and an unfavorable prognosis for the course of the disease [21,22].

Overexpression of miRNA-155, which manifests as a decrease in the level of taurine and an increase in the level of oxidative stress, is often found in breast tumor tissue and negatively affects survival and chemosensitivity (through the FOXO3a gene) of tumor cells, while reduced expression this miRNA can enhance cellular chemosensitivity and apoptosis. The activity of miRNA-155 [23] is necessary for the normal functioning of cells; an increase in the expression of this miRNA has been noted in autoimmune diseases and various forms of cancer. In addition, miRNA-155 is associated with the estrogen-positive status of tumors and can potentially serve as a diagnostic marker.

miRNA-221/222 is classified as an oncogenic miRNA, the overexpression of which in different types of tumors leads to increased cell proliferation, inhibition of apoptosis, and induction of angiogenesis. The suppression and low expression level of miRNA-221/222 in breast tumors correlate with the positive status of estrogen receptors and a more favorable prognosis of the disease.

According to a previous report [24], miRNA-205 is a tumorigenesis suppressor; it induces apoptosis and inhibits the growth and invasion of tumor cells.

Several miRNAs (21, 155, 221, and 222) have been studied in patients diagnosed with breast fibroadenoma. The authors of these studied noted a ten-fold increase in the number of miRNAs that induce apoptosis and cell growth. Another study on the role of miRNAs 137, 199, and others provided an informative analysis of the body’s resistance to tumor aggression [25,26,27] for early cancer diagnosis.

Breast cancers have complex phenotypes, characterized by a large number of cellular and biomolecular formations. Biological pathways have been successfully used to reveal some heterogeneity in phenotypes between disease states, while gene networks have been used to study large-scale regulatory patterns. Ultimately, biological processes are carried out by proteins and their complexes. Therefore, as demonstrated in a recent study [28], the profiling of breast cancers can be extended by analyzing open proteomic data along with gene expression.

It is known that miRNAs are aberrantly expressed in the serum, tissues, and peripheral blood mononuclear cells of cancer patients and can serve as potential non-invasive diagnostic markers of breast cancer. In a previous study [29], differentially expressed miRNAs were identified using next generation sequencing in breast cancer patients and healthy people of the same age. Four miRNAs—three (miR-24-2-5p, miR3609, and miR-664b-3p) downregulated and one (miR-192-5p) overexpressed—were identified as potential biomarkers for patients with locally advanced breast cancer.

Nucleic acid sequence-based amplification (NASBA) is a specific single-stranded RNA fragment amplification technique that is useful for highly sensitive miRNA detection. In a study by Karasawa and colleagues [30], the authors developed a new miRNA analytical system by combining NASBA and chemiluminescence. Since the NASBA reaction is carried out at a constant temperature and detection by the chemiluminescence reaction does not require a light source, these methods can be combined for miRNA amplification. This combined miRNA detection method may be useful for the future development of compact point-of-care testing systems.

With regard to other forms of cancer, miRNAs, which can be used as stand-alone biomarkers or integrated into molecular signatures of clinical interest, are considered ideal for diagnostic purposes and accurate molecular classification of kidney tumors. In addition, miRNAs could serve as prognostic biomarkers in patients with renal cell carcinomas, facilitating the prediction of relapse-free and overall survival of patients, thus reducing over- and/or under-treatment. miRNAs can also be used as predictors of a patient’s response to targeted therapy with tyrosine kinase inhibitors, facilitating the decision-making process for selecting an appropriate treatment plan [31,32].

Currently, cancer diagnosis is undergoing a paradigm shift by incorporating molecular biomarkers, such as DNA, RNA, miRNA, and proteins, into the routine diagnostic panel. In recent years, researchers have developed deep learning-based methods for cancer diagnosis. To overcome the superclass problem, an improved generative adversarial network has been proposed, optimized using the Mayfly optimization algorithm [33]. The enhanced generative adversarial network is a combination of the deep convolutional generative adversarial network (DCG) and the modified convolutional neural network (MCNN). Along with this new algorithm, the algorithms CMiRNA-BC-CNN, which uses a new representation algorithm and evolutionary deep learning, and CMiRNA-BC-GCNN, which uses multi-ohm data with graph convolutional networks, already exist for the classification of cancer miRNA biomarkers. Further neural networks with an optimization process are being developed to select the correct miRNA data [34,35].

## 5. Conclusions

The combined use of the classical methods of MMG and ultrasound exams with MWR, meta-omics, and miRNA with neural networks for early breast cancer screening will make it possible to identify pathologies that will most likely turn into cancer in the future. When used alone, these novel methods indicated slightly raised probabilities of cancer development in patients with high-risk malignancies, while the combination of these methods gave clear indications of these malignancies. This novel approach will make it possible to prescribe at-risk individuals with onco-prophylactic complexes to regress obligate precancers, thereby reducing the number of precancers that develop into breast cancer.

## Figures and Tables

**Table 1:** miRNA expression at the initial investigation in patient P. Changes in the quantitative indicators (copies/μL) between “Norm” and “Pathology” of 1.5–2 times indicate a slight increase (risk factor = 1), 2–5 times indicate a moderate increase (risk factor = 2), and 5 times or above indicate a significant increase (risk factor = 3). The threshold for each miRNA was determined by precedent statistics from literature. Concentration values are presented as log base 2 miRNA (copies/μL).

N	Marker	Norm	Pathology	Fold Change
1	Hsa-miR-155	28,294	202,238	7.1
2	Hsa-miR-196a-3p	104,809	324,654	3.1
3	Hsa-miR-222-3p	159,928	88,908	0.6
4	Hsa-let-7a-5p	13,327,568	2,665,531	0.2
5	Micro-196a-2	1,665,957	333,191	0.2
6	Hsa-miR-106a-5p	380,633	76,126	0.2
7	Has-miR-21-5p	22,209,402	111,047,010	5.0
8	Hsa-miR-137	555,235,050	111,047,010	0.2

**Table 2:** miRNA expression in patient P at the initial investigation.

N	Marker	Expression
1	Hsa-miR-155	Norm
2	Hsa-miR-196a-3p	Norm
3	Hsa-miR-222-3p	Slight increase
4	Hsa-let-7a-5p	Slight increase
5	Micro-196a-2	Norm
6	Hsa-miR-106a-5p	Slight increase
7	Has-miR-21-5p	Norm
8	Hsa-miR-137	Norm

**Table 3:** miRNA expression in patient P after 6 months.

N	Marker	Expression
1	Hsa-miR-155	Norm
2	Hsa-miR-196a-3p	Norm
3	Hsa-miR-222-3p	Pronounced increase—3
4	Hsa-let-7a-5p	Pronounced increase—3
5	Micro-RNA-196a-2	Norm
6	Hsa-miR-106a-5p	Slight increase—1
7	Has-miR-21-5p	Norm
8	Hsa-miR-137	Norm

## Data Availability

The data presented in this study are available on request from the corresponding author. The data are not publicly available due to ethics restrictions.
